# Ethane-1,2-diammonium naphthalene-1,5-disulfonate

**DOI:** 10.1107/S1600536809039762

**Published:** 2009-10-07

**Authors:** Zhi-Biao Zhu, Shan Gao, Seik Weng Ng

**Affiliations:** a College of Chemistry and Materials Science, Heilongjiang University, Harbin 150080, People’s Republic of China; b Department of Chemistry, University of Malaya, 50603 Kuala Lumpur, Malaysia

## Abstract

In the crystal structure of the title salt, C_2_H_10_N_2_^2+^·C_10_H_6_O_6_S_2_^2−^, both the cation and anion lie on special positions of 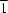 site symmetry. These are linked by N—H⋯O and N—H⋯(O,O) hydrogen bonds, forming a layer structure.

## Related literature

For the crystal structures of ammonium 1,5-naphthalene­disulfonates, see, for example: Russel *et al.* (1997 ▶); Sakwa & Wheeler (2003 ▶); Zhang *et al.* (2004 ▶). 
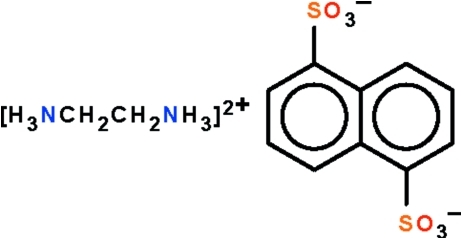


## Experimental

### 

#### Crystal data

C_2_H_10_N_2_^2+^·C_10_H_6_O_6_S_2_^2−^
*M* *_r_* = 348.39
Monoclinic, 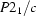

*a* = 11.188 (7) Å
*b* = 8.230 (4) Å
*c* = 8.492 (6) Å
β = 100.19 (3)°
*V* = 769.6 (8) Å^3^
*Z* = 2
Mo *K*α radiation
μ = 0.38 mm^−1^
*T* = 293 K
0.31 × 0.27 × 0.23 mm

#### Data collection

Rigaku R-AXIS RAPID IP diffractometer
Absorption correction: multi-scan (*ABSCOR*; Higashi, 1995 ▶) *T*_min_ = 0.892, *T*_max_ = 0.919
7310 measured reflections
1759 independent reflections
1599 reflections with *I* > 2σ(*I*)
*R*_int_ = 0.012

#### Refinement

*R*[*F* ^2^ > 2σ(*F* ^2^)] = 0.031
*wR*(*F* ^2^) = 0.097
*S* = 1.06
1759 reflections
112 parameters
3 restraints
H atoms treated by a mixture of independent and constrained refinement
Δρ_max_ = 0.42 e Å^−3^
Δρ_min_ = −0.23 e Å^−3^

### 

Data collection: *RAPID-AUTO* (Rigaku, 1998 ▶); cell refinement: *RAPID-AUTO*; data reduction: *CrystalClear* (Rigaku/MSC, 2002 ▶); program(s) used to solve structure: *SHELXS97* (Sheldrick, 2008 ▶); program(s) used to refine structure: *SHELXL97* (Sheldrick, 2008 ▶); molecular graphics: *X-SEED* (Barbour, 2001 ▶); software used to prepare material for publication: *publCIF* (Westrip, 2009 ▶).

## Supplementary Material

Crystal structure: contains datablocks global, I. DOI: 10.1107/S1600536809039762/xu2622sup1.cif

Structure factors: contains datablocks I. DOI: 10.1107/S1600536809039762/xu2622Isup2.hkl

Additional supplementary materials:  crystallographic information; 3D view; checkCIF report

## Figures and Tables

**Table 1:** Hydrogen-bond geometry (Å, °)

*D*—H⋯*A*	*D*—H	H⋯*A*	*D*⋯*A*	*D*—H⋯*A*
N1—H11⋯O1	0.87 (1)	2.31 (1)	3.052 (2)	144 (2)
N1—H11⋯O2	0.87 (1)	2.38 (1)	3.137 (3)	147 (2)
N1—H12⋯O1^i^	0.87 (1)	1.93 (1)	2.7800 (19)	164 (2)
N1—H13⋯O2^ii^	0.86 (1)	1.94 (1)	2.790 (2)	171 (2)

Symmetry codes: (i) 
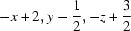
; (ii) 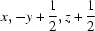.

**Fig. 1.**
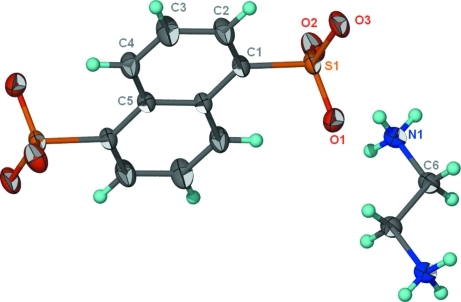
Thermal ellipsoid plot (Barbour, 2001) of [C12H10N2] [C10H6O6S2] at the 50% probability level; hydrogen atoms are drawn as spheres of arbitrary radius.

## supplementary crystallographic information

### Experimental

To an aqueous solution of sodium naphthalene-1,5-disulfonate (0.58 g, 2 mmol) and ethylenediamine was added cobalt diacetate trihydrate (0.46 g, 2 mmol). The mixture was stirred for 15 min and then filtered. Colorless crystals of the organic salt separated from the solution after a few days. CH&N elemental analysis. Calc. for C_10_H_16_N_2_O_6_S_2_: C 37.03, H 4.97, N 8.64%; found: C 37.06, H 4.91, N 8.68%.

### Refinement

Carbon-bound H-atoms were placed in calculated positions (C—H 0.93 to 0.97 Å) and were included in the refinement in the riding model approximation, with *U*(H) set to 1.2*U*(C). The ammonium H-atoms were refined with a distance restraint of N–H 0.86±0.01 Å; their temperature factors were refined.

### Crystal data

**Table:**

C_2_H_10_N_2_^2+^·C_10_H_6_O_6_S_2_^2^^−^	*F*(000) = 364
*M**_r_* = 348.39	*D*_x_ = 1.503 Mg m^−^^3^
Monoclinic, *P*2_1_/*c*	Mo *K*α radiation, λ = 0.71073 Å
Hall symbol: -P 2ybc	Cell parameters from 6820 reflections
*a* = 11.188 (7) Å	θ = 3.1–27.5°
*b* = 8.230 (4) Å	µ = 0.38 mm^−^^1^
*c* = 8.492 (6) Å	*T* = 293 K
β = 100.19 (3)°	Prism, colorless
*V* = 769.6 (8) Å^3^	0.31 × 0.27 × 0.23 mm
*Z* = 2	

### Data collection

**Table:**

Rigaku R-AXIS RAPID IP diffractometer	1759 independent reflections
Radiation source: fine-focus sealed tube	1599 reflections with *I* > 2σ(*I*)
graphite	*R*_int_ = 0.012
ω scans	θ_max_ = 27.5°, θ_min_ = 3.1°
Absorption correction: multi-scan (*ABSCOR*; Higashi, 1995)	*h* = −14→14
*T*_min_ = 0.892, *T*_max_ = 0.919	*k* = −9→10
7310 measured reflections	*l* = −11→11

### Refinement

**Table:**

Refinement on *F*^2^	Primary atom site location: structure-invariant direct methods
Least-squares matrix: full	Secondary atom site location: difference Fourier map
*R*[*F*^2^ > 2σ(*F*^2^)] = 0.031	Hydrogen site location: inferred from neighbouring sites
*wR*(*F*^2^) = 0.097	H atoms treated by a mixture of independent and constrained refinement
*S* = 1.06	*w* = 1/[σ^2^(*F*_o_^2^) + (0.0652*P*)^2^ + 0.1775*P*] where *P* = (*F*_o_^2^ + 2*F*_c_^2^)/3
1759 reflections	(Δ/σ)_max_ = 0.001
112 parameters	Δρ_max_ = 0.42 e Å^−^^3^
3 restraints	Δρ_min_ = −0.23 e Å^−^^3^

### Fractional atomic coordinates and isotropic or equivalent isotropic displacement parameters (Å^2^)

**Table:**

	*x*	*y*	*z*	*U*_iso_*/*U*_eq_	
S1	0.80614 (3)	0.49980 (4)	0.49518 (4)	0.02704 (14)	
O1	0.83952 (9)	0.58324 (13)	0.64845 (12)	0.0385 (3)	
O2	0.82188 (9)	0.32459 (13)	0.51712 (14)	0.0415 (3)	
O3	0.86701 (9)	0.56466 (15)	0.37277 (13)	0.0406 (3)	
N1	0.98833 (11)	0.32604 (14)	0.85576 (14)	0.0297 (3)	
C1	0.64836 (11)	0.53300 (16)	0.43172 (15)	0.0262 (3)	
C2	0.61199 (13)	0.6030 (2)	0.28556 (18)	0.0388 (3)	
H2	0.6693	0.6333	0.2240	0.047*	
C3	0.48746 (14)	0.6294 (2)	0.22779 (19)	0.0450 (4)	
H3	0.4629	0.6766	0.1278	0.054*	
C4	0.40295 (12)	0.58634 (18)	0.31707 (17)	0.0349 (3)	
H4	0.3212	0.6047	0.2771	0.042*	
C5	0.43711 (11)	0.51406 (14)	0.46991 (16)	0.0239 (3)	
C6	1.04788 (12)	0.44939 (17)	0.96990 (16)	0.0310 (3)	
H6A	1.0988	0.5190	0.9175	0.037*	
H6B	1.0990	0.3962	1.0593	0.037*	
H11	0.9543 (15)	0.367 (2)	0.7649 (14)	0.041 (5)*	
H12	1.0407 (14)	0.254 (2)	0.835 (2)	0.048 (5)*	
H13	0.9362 (13)	0.2720 (19)	0.8978 (19)	0.041 (5)*	

### Atomic displacement parameters (Å^2^)

**Table:**

	*U*^11^	*U*^22^	*U*^33^	*U*^12^	*U*^13^	*U*^23^
S1	0.0183 (2)	0.0338 (2)	0.0301 (2)	0.00095 (10)	0.00700 (14)	0.00333 (11)
O1	0.0296 (5)	0.0519 (6)	0.0336 (5)	−0.0094 (4)	0.0046 (4)	−0.0029 (4)
O2	0.0324 (5)	0.0361 (6)	0.0585 (7)	0.0095 (4)	0.0152 (5)	0.0056 (5)
O3	0.0255 (5)	0.0610 (7)	0.0380 (6)	−0.0009 (5)	0.0130 (4)	0.0098 (5)
N1	0.0320 (6)	0.0295 (6)	0.0288 (5)	0.0020 (5)	0.0085 (4)	0.0005 (4)
C1	0.0195 (6)	0.0303 (6)	0.0293 (6)	0.0013 (5)	0.0058 (4)	0.0031 (5)
C2	0.0265 (6)	0.0556 (9)	0.0362 (7)	0.0008 (6)	0.0110 (5)	0.0172 (6)
C3	0.0312 (7)	0.0663 (10)	0.0371 (8)	0.0055 (7)	0.0056 (6)	0.0266 (7)
C4	0.0230 (6)	0.0473 (8)	0.0336 (7)	0.0040 (6)	0.0027 (5)	0.0126 (6)
C5	0.0207 (6)	0.0249 (6)	0.0268 (6)	0.0012 (4)	0.0060 (5)	0.0026 (4)
C6	0.0294 (7)	0.0322 (6)	0.0325 (7)	0.0010 (6)	0.0087 (5)	−0.0031 (5)

### Geometric parameters (Å, °)

**Table:**

S1—O3	1.4421 (12)	C2—H2	0.9300
S1—O1	1.4606 (14)	C3—C4	1.359 (2)
S1—O2	1.4606 (13)	C3—H3	0.9300
S1—C1	1.7733 (17)	C4—C5	1.417 (2)
N1—C6	1.4784 (19)	C4—H4	0.9300
N1—H11	0.867 (9)	C5—C5^i^	1.428 (3)
N1—H12	0.870 (9)	C5—C1^i^	1.4300 (18)
N1—H13	0.860 (9)	C6—C6^ii^	1.516 (3)
C1—C2	1.363 (2)	C6—H6A	0.9700
C1—C5^i^	1.4300 (18)	C6—H6B	0.9700
C2—C3	1.410 (2)		
			
O3—S1—O1	112.91 (8)	C3—C2—H2	120.0
O3—S1—O2	113.31 (7)	C4—C3—C2	120.52 (13)
O1—S1—O2	110.14 (7)	C4—C3—H3	119.7
O3—S1—C1	107.19 (7)	C2—C3—H3	119.7
O1—S1—C1	106.39 (7)	C3—C4—C5	121.23 (13)
O2—S1—C1	106.38 (6)	C3—C4—H4	119.4
C6—N1—H11	113.1 (12)	C5—C4—H4	119.4
C6—N1—H12	110.9 (13)	C4—C5—C5^i^	118.99 (14)
H11—N1—H12	106.8 (17)	C4—C5—C1^i^	123.28 (12)
C6—N1—H13	110.1 (12)	C5^i^—C5—C1^i^	117.73 (15)
H11—N1—H13	110.4 (16)	N1—C6—C6^ii^	109.58 (15)
H12—N1—H13	105.3 (17)	N1—C6—H6A	109.8
C2—C1—C5^i^	121.56 (12)	C6^ii^—C6—H6A	109.8
C2—C1—S1	117.55 (10)	N1—C6—H6B	109.8
C5^i^—C1—S1	120.89 (10)	C6^ii^—C6—H6B	109.8
C1—C2—C3	119.96 (13)	H6A—C6—H6B	108.2
C1—C2—H2	120.0		
			
O3—S1—C1—C2	2.51 (14)	C5^i^—C1—C2—C3	−0.5 (2)
O1—S1—C1—C2	123.56 (13)	S1—C1—C2—C3	178.79 (14)
O2—S1—C1—C2	−119.01 (13)	C1—C2—C3—C4	0.4 (3)
O3—S1—C1—C5^i^	−178.25 (11)	C2—C3—C4—C5	0.0 (3)
O1—S1—C1—C5^i^	−57.19 (13)	C3—C4—C5—C5^i^	−0.2 (2)
O2—S1—C1—C5^i^	60.24 (12)	C3—C4—C5—C1^i^	179.91 (15)

Symmetry codes: (i) −*x*+1, −*y*+1, −*z*+1; (ii) −*x*+2, −*y*+1, −*z*+2.

### Hydrogen-bond geometry (Å, °)

**Table:**

*D*—H···*A*	*D*—H	H···*A*	*D*···*A*	*D*—H···*A*
N1—H11···O1	0.87 (1)	2.31 (1)	3.052 (2)	144 (2)
N1—H11···O2	0.87 (1)	2.38 (1)	3.137 (3)	147 (2)
N1—H12···O1^iii^	0.87 (1)	1.93 (1)	2.7800 (19)	164 (2)
N1—H13···O2^iv^	0.86 (1)	1.94 (1)	2.790 (2)	171 (2)

Symmetry codes: (iii) −*x*+2, *y*−1/2, −*z*+3/2; (iv) *x*, −*y*+1/2, *z*+1/2.
